# Genome-Wide Association-Based Identification of Alleles, Genes and Haplotypes Influencing Yield in Rice (*Oryza sativa* L.) Under Low-Phosphorus Acidic Lowland Soils

**DOI:** 10.3390/ijms252111673

**Published:** 2024-10-30

**Authors:** M. James, Wricha Tyagi, P. Magudeeswari, C. N. Neeraja, Mayank Rai

**Affiliations:** 1 School of Crop Improvement, College of Post Graduate Studies in Agricultural Sciences, Central Agricultural University (Imphal), Umiam 793103, Meghalaya, India; mailmejames.2009@gmail.com (M.J.); wricha.tyagi@icrisat.org (W.T.); magudeeswarip35@gmail.com (P.M.); 2 Research Program—Accelerated Crop Improvement (ACI), International Crops Research Institute for the Semi-Arid Tropics, Patancheru 502324, Telangana, India; 3 ICAR—Indian Institute of Rice Research, Hyderabad 500030, Telangana, India; cnneeraja@gmail.com; 4 Post Graduate College of Agriculture, Dr. Rajendra Prasad Central Agricultural University (RPCAU), Samastipur 848125, Bihar, India

**Keywords:** peak SNP, acidic soils, rice, GWAS, haplotype

## Abstract

Rice provides poor yields in acidic soils due to several nutrient deficiencies and metal toxicities. The low availability of phosphorus (P) in acidic soils offers a natural condition for screening genotypes for grain yield and phosphorus utilization efficiency (PUE). The objective of this study was to phenotype a subset of indica rice accessions from 3000 Rice Genome Project (3K-RGP) under acidic soils and find associated genes and alleles. A panel of 234 genotypes, along with checks, were grown under low-input acidic soils for two consecutive seasons, followed by a low-P-based hydroponic screening experiment. The heritability of the agro-morphological traits was high across seasons, and Ward’s clustering method identified 46 genotypes that can be used as low-P-tolerant donors in acidic soil conditions. Genotypes ARC10145, RPA5929, and K1559-4, with a higher grain yield than checks, were identified. Over 29 million SNPs were retrieved from the Rice SNP-Seek database, and after quality control, they were utilized for a genome-wide association study (GWAS) with seventeen traits. Ten quantitative trait nucleotides (QTNs) for three yield traits and five QTNs for PUE were identified. A set of 34 candidate genes for yield-related traits was also identified. An association study using this indica panel for an already reported 1.84 Mbp region on chromosome 2 identified genes *Os02g09840* and *Os02g08420* for yield and PUE, respectively. A haplotype analysis for the candidate genes identified favorable allelic combinations. Donors carrying the superior haplotypic combinations for the identified genes could be exploited in future breeding programs.

## 1. Introduction

Rice (*Oryza sativa*) is the most consumed food crop in Asia. On average, 700 and 500 million tonnes of paddy are respectively produced and consumed annually worldwide. Around 50% of the world [1] and 34.5% of Indian [2] cultivated soils are acidic in nature. Rice plants grown in acid soils show a reduced grain yield and plant biomass [3]. Strong to moderate (pH 4.5–6.5) acidic soils are predominant in northeast India, especially the rainfed lowlands of Meghalaya [4], and phosphorous (P) gets fixed into compounds that are unavailable to plants. Traditional practices of low fertilizer application over the years also make the soil deficient in available P, more than 60% of which that is taken up in cereal crops is translocated into grains [5]. Therefore, developing low-P-tolerant genotypes is the best sustainable solution for problematic acidic soils [6].

Apart from low P, acidic soils undergo other stresses like aluminum, iron and manganese toxicity [7] and calcium and magnesium deficiency. Hence, screening specifically for low-P tolerance in acidic soils is confounded by other stresses. In early growth stages, hydroponics is a potential method to screen a large number of accessions in a short time. Mostly, for low-P screening, the Yoshida nutrient solution is used, and traits like root length, root and shoot biomass and PUE (phosphorous use efficiency) are studied [8,9,10]. However, traits from hydroponics cannot usually be used directly for the selection of genotypes. Therefore, traits used for hydroponics screening, coupled with yield and related traits under low-P field conditions, should be considered to identify potentially high-yielding tolerant genotypes.

Although nineteen genes, thirteen mutants and many QTLs (quantitative trait loci) are reported to govern low-P tolerance in rice [11], *PSTOL1* (Phosphorous Starvation Tolerance 1) is the only gene that is deployed in breeding programs through marker-assisted selection [12]. Additionally, genetic background plays a major role in determining low-P tolerance in addition to the gene itself. A diverse natural population like 3K RDP (3024, Rice Diversity Panel) is an ideal genomic resource for identifying SNPs (single nucleotide polymorphism) associated with low-P tolerance across diverse genetic backgrounds, and 3K RDP was established by combining accessions from the International Rice Research Institute (IRRI) and China National Crop Gene Bank (CNCGB) [13]. All of these genotypes were sequenced in 2014, and 32 million SNPs among these genotypes are publicly available in the SNP-Seek IRRI database [14].

A GWAS (genome-wide association study) associates polymorphic SNPs with the phenotype of diverse populations and thus serves as a potential tool in identifying new genes with a high resolution across the genome in a single study [15]. Many GWAS studies using a subset of genotypes from the 3K panel for traits like agronomic traits [15], seedling vigor [16], drought tolerance [17], sulfur tolerance [18], seed storability [19], weedy traits [20], low soil fertility tolerance [21] and nitrogen deficiency tolerance are reported [22]. This dataset has also been used for an in silico polymorphism survey of loci reported with various traits using a small set of contrasting genotypes [10,23]. In the current study, a subset of 3K RDP was grown under low-input acidic soils with low available P for two years, and yield and component traits were recorded. Additionally, a low-P-stress hydroponics experiment was also conducted. GWAS led to the identification of diverse tolerant genotypes, peak SNPs associated with low-P tolerance and superior haplotypes imparting a higher yield under low-P conditions. Previously, we had mapped a 1.847 Mb region on chromosome 2 that is associated with a high yield under low-P acidic soils, using a biparental mapping population [10]. In this region, several QTLs reported for various yield-related traits, including drought-tolerant yield QTL (qDTY2.2) [24], are located. Hence, we aimed to narrow this region down and detect associated SNPs using the same resource and approach.

## 2. Results

### 2.1. Nature of the Soil and Population Structure of the Genotypes Grown in Acidic Soils

Soil samples were taken at five random places within the ten blocks before transplanting for the two consecutive years, 2020 and 2021. The soil pH ranged between 5.5 and 6, and the average P content was less than 9 kg per hectare. The 234 genotypes of the 3K RDP (3024 rice diversity panel) belonged to eight subpopulation groups, namely indica (ind1A, indx, ind2, ind3), aus, japonica, temperate, tropical, subtropical, aromatic and admixtures; among them, the *indica* type was the highest (*ind2*—36%, *indx*—28.4%) followed by the *aus*-type subgroup (19.1%) (Supplementary Figure S1A). The 34 lakh SNP data were used to perform population structure, kinship and PCA evaluations, and the results showed the presence of three distinct groups (Supplementary Figure S1B–F). The grouping confirmed the 3K MDS (MultiDimensional Scaling) plot downloaded from the SNP-Seek IRRI database [14]. The grouping was able to distinguish *indica*- and *aus*-type genotypes that are highlighted in the representative check genotypes Swarna and Kasalath that belong to the *indica* and *aus* types, respectively (Supplementary Figure S1C,D).

### 2.2. Superior Genotypes for Yield Under Low-P Field Conditions and in Hydroponics Experiment

Ten agro-morphological (TN: Tiller number; PL: Panicle length; SPP: spikelets per panicle; FGPP: filled grains per panicle; SF: spikelet fertility percentage; BY: biological yield; GYPP: grain yield per plant; DM: dry matter; PY: plot yield; HI: harvest index) and two inorganic phosphorus (Pi) estimation-related traits (PUE: phosphorus utilization efficiency; PC: phosphorous concentration) were studied in both seasons (Supplementary Figure S2). Variance components were obtained from the restricted maximum likelihood (REML) method based on a combined environment analysis for field- and Pi-related traits (Supplementary Table S2). There were significant variations among the genotypes for all the traits studied except for SF, HI, PC and PUE, and there was no significant variation between the two seasons. The heritability of the studied agro-morphological traits was high, whereas the two Pi-related traits, PC and PUE, showed low heritability. The phenotypic BLUP (best linear unbiased prediction) values of the ten agro-morphological and two Pi estimation-related traits were calculated (Supplementary Table S3) and used for all subsequent analyses.

**Figure 1:**
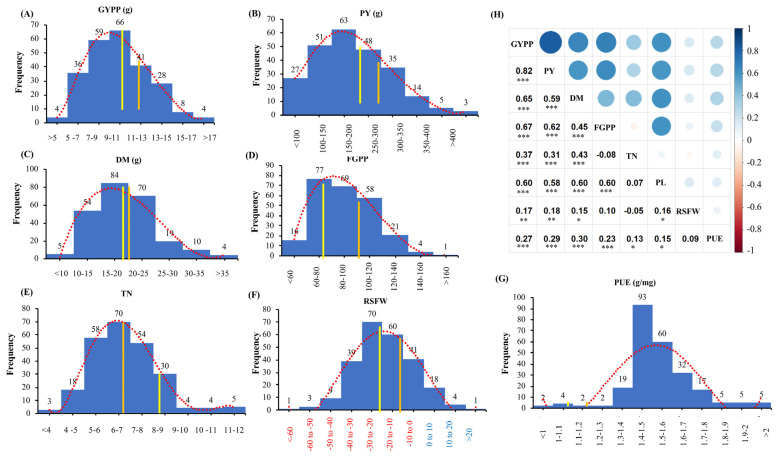
Distribution frequency using BLUP values for seven traits for 237 rice genotypes. (**A**) GYPP (grain yield per plant); (**B**) PY (plot yield); (**C**) DM (dry matter); (**D**) FGPP (filled grains per panicle); (**E**) TN (tiller number); (**F**) RSFW (relative shoot fresh weight; red and blue numbers on x axis represent positive values, respectively); (**G**) PUE (phosphorus utilization efficiency). The yellow and orange bars represent the performance of the checks Kasalath (*aus*) and Swarna (*indica*), respectively, while the dotted red line is a polynomial curve indicating the normal distribution for each trait. (**H**) Heatmap depicting the correlation between the eight traits. Pearson’s r values are given on the left, and a corresponding heatmap is shown on the right, with blue and red colors indicating positive and negative correlations, respectively. Significant values are indicated as * (*p* < 0.05), ** (*p* < 0.01) and *** (*p* < 0.001).

The frequency distribution curve for the important traits showed a normal distribution (Figure 1A–G), and the performance of the common checks like Kasalath and Swarna were nearly average for all the traits. The range of GYPP was from 3.1 to 24 g in kharif, 2020 and 2.4 to 20 g in kharif, 2021. In a combined-season analysis, the data were normalized, and the range was between 4.5 and 18 g (Figure 1A). The genotypes ARC10145, RPA5929, K1559-4 and ARC10799 had the highest GYPP of more than 17 g. Similarly, PY ranged from 63 to 563 g (Figure 1B), and the genotypes Aus paddy and ARC10145 were the highest yielders in the combined season analysis, yielding more than 400 g. In the combined analysis, genotypes Bir bahadur, Code no 31225, CN 44-40-7 and Sons aus exhibited more than ten tillers. Genotypes like ARC14299, Chile boro and NCS 964C exhibited more than 35 g and 50 g of DM and BY, respectively. Checks like Shasharang, DRR Dhan48 and CAUS105 were the high-yielding checks in both seasons (>20 g). The P concentration was between 0.7 and 1.1 mg of Pi per gram of flag leaf at harvest, and PUE ranged between 1 and 3 g per milligram of Pi (Figure 1G). The observed PUE was highest in the genotypes DRR Dhan 48, ARC 15,088 and Makarandasail.

Five traits (RRL: relative root length; RSFW: relative shoot fresh weight; RRFW: relative root fresh weight; RTFW: relative total fresh weight; RTDW: relative total dry weight) were studied for the genotypes grown in the hydroponics condition; average values were calculated (Supplementary Table S5) to perform a two-way ANOVA. The genotypes showed significant differences in both the control and treatment conditions for all the traits studied (Supplementary Table S4). The genotypes Labra, ARC 12124, Lakha kuar and JC149 exhibited a significantly longer root length in the low-P treatment in comparison to the control (Supplementary Figure S2D). The genotypes Zinco rice ms, Poongar and ARC15163 had a high root fresh weight in the treatment (Supplementary Figure S2E). For the rest of the three traits, there was no genotype that had significantly higher values in the low-P treatment. Banikat, ARC 10939, dudre, ARC 14901, Lanjali and seventeen other genotypes, including check SD, exhibited positive RSFW, which is desirable.

### 2.3. Correlation Analysis and Identifying Tolerant and Susceptible Genotypes

Overall, seventeen different traits were measured, and a correlation matrix was plotted (Supplementary Figure S3A). Our previous data for yield and low-P tolerance under acidic soils suggest that eight traits, namely TN, PL, FGPP, DM, GYPP, PY, PUE and RSFW, are important contributors to yield under a low-P soil condition. A correlation analysis for these eight traits revealed that GYPP and PY were significant and positively associated with all the other traits (Figure 1). Traits like PL and DM showed a significant positive correlation with PUE and RSFW, whereas the traits TN and FGPP had a positive correlation with RSFW. The Euclidean distance clustering (Ward’s method) based on these traits resulted in three clusters (Supplementary Figure S3B). The genotypes belonging to cluster I were poor yielders, whereas cluster II had high-yielding genotypes. Cluster III had average-performing genotypes, including the checks Kasalath and Swarna. Cluster II included checks like Shasharang, DRR Dhan48 and other genotypes that can be used to breed for low-P tolerance [25] and high yield in acidic soils.

### 2.4. Identification of Desirable Haplotypes for Yield in Low-P Acidic Soils for the 1.847 Mb Region on Chromosome 2

A 1.847 Mb region associated with low-P tolerance was previously identified on chromosome 2 in a biparental population derived from SD and Chakhao Poireiton [10]. The region spanned from *Os02g07780* at 4,073,883 bp to *Os02g11130* at 5,967,668 bp with 281 annotated loci. The entire region is of 18,93,785 bp length with reference to Nipponbare, and a total of 21,702 SNPs (single nucleotide polymorphisms) were obtained, which, on filtering, yielded 16,090 polymorphic SNPs. A linear mixed-model approach in the EMMA eXpedited software (version emmax-beta-07Mar2010) (EMMAX)-based association study for this region with respect to eight traits (TN, PL, FGPP, DM, GYPP, PY, PUE and RSFW) led to the detection of 71 peak SNPs in 25 annotated loci, with ten loci having SNPs in exons. The genes present in the local LD (linkage disequilibrium) blocks were extracted, and a haplotype analysis was done for 235 genotypes (Supplementary Figure S4). Three loci, namely *Os02g09820* (zinc finger C3HC4), *Os02g09830* (*bZIP* transcription factor) and *Os02g09840* (serine/threonine-protein phosphatase 2A), included SNPs that were associated with GYPP and PY (Supplementary Table S6). Based on closely spaced SNPs within an LD block, *Os02g09840* was considered the candidate gene (Supplementary Figure S4D). A haplotype analysis including two nonsynonymous SNPs within the genes showed significant differences between haplotypes for both PY and GYPP (Supplementary Figure S4E–G). The SNP at the 5,073,629th position causes a change in amino acid from alanine (C) to glycine (G) with a sift score of ‘0’ meaning the change might have a negative effect, and at the 5,073,665th position, there was a change in isoleucine (G) to threonine (A). Both of these SNPs were in PP2A regulatory subunit B, EF-hand domain. Among the three low-P-tolerant checks used in this study for which genotypic information is available, the desirable haplotype1 was present only in IAC47. Peak SNPs were also detected for PUE in this region, and *Os02g08420* (cinnamoyl CoA reductase) was considered the candidate gene (Supplementary Figure S4C). A haplotype analysis revealed four haplotypes, with haplotype 1 having significantly superior PUE (Supplementary Figure S4H–I). The other candidate genes, *Os02g09820* and *Os02g09830*, also had nonsynonymous SNPs that were associated with a significant increase in GYPP and PY. Three loci, *Os02g10750*, Os02g10920 and *Os02g11000*, coding for CBL-interacting protein kinase, zinc finger protein and agenet domain-containing protein, respectively, were detected for DM and PL (Supplementary Table S6).

### 2.5. Genome-Wide Association Study (GWAS) and Identification of Candidate Genes for Yield Under Lowland Acid Soil

**Figure 2:**
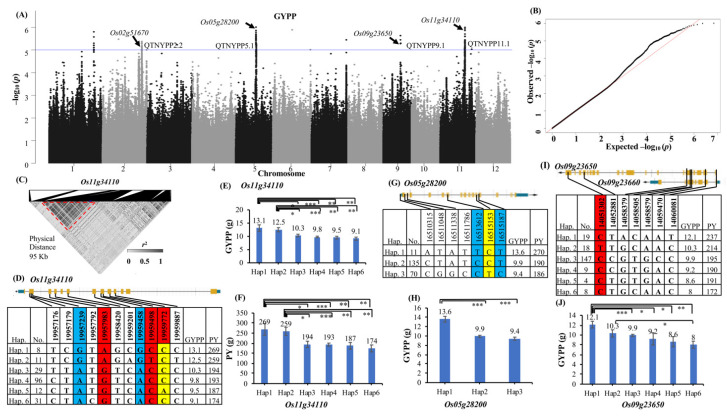
Identification of candidate genes associated with grain yield per plant (GYPP) in low-P field conditions. (**A**) Manhattan plot of GYPP with EMMAX model showing QTNs and associated candidate genes (highlighted with arrows). Horizontal lines in the Manhattan plots indicate the genome-wide thresholds -log *P* values of 5 (blue) and 7.5 (red). (**B**) Q–Q plot for GYPP. The dashed (red) line in Q–Q plot represents significance threshold, whereas black dots represent observed values. (**C**) Zoomed-in SNP likelihood LD (linkage disequilibrium) heatmap showing the peak SNP and the position of candidate gene *Os11g34110* within the dotted red triangle. Gene structure of candidate genes—(**D**) *Os11g34110*, (**G**) *Os05g28200* and (**I**) *Os09g23650*—with haplotype analysis of peak SNPs. The orange, blue and white colors represent exons, UTR and introns, respectively. Nonsynonymous SNPs are in bold; the blue-, red- and yellow-colored columns represent peak, deleterious SNP (SIFT score < 0.05) and the splice variant, respectively. The average value for a particular haplotype (hap.) for GYPP and PY, along with the number of genotypes (no.), is indicated. Phenotypic variation among haplotypes for GYPP and PY with significant values (*t*-test) indicated as * (*p* < 0.05), ** (*p* < 0.01) and *** (*p* < 0.001), respectively, for candidate genes—(**E**) and (**F**) *Os11g34110*, (**H**) *Os05g28200* and (**J**) *Os09g23650*.

Four quantitative trait nucleotides (QTNs) for grain yield per plant (GYPP) in chromosomes 2, 5, 9 and 11 were identified (Figure 2 and Table 1).

The QTN for the grain yield per plant on chromosome 11 (QTNGYPP11.1) spanned 95 kb in length and included only one annotated gene, *Os11g34110*, encoding a heparan-alpha-glucosaminide N-acetyltransferase protein. There were 16 significant peak SNPs present with eleven nonsynonymous SNPs leading to six haplotypes (Figure 2D). There was a decreasing trend in yield across haplotypes (Figure 2E); haplotype 1 had the highest GYPP (13.1 ± 1.2 g), whereas haplotype 6 had the lowest GYPP (9.1 ± 0.6 g). There was a difference of 95 g between the two haplotypes for PY (Figure 2F). Desirable haplotype1 was present in eight genotypes, which represent 73% of *temperate* and 44% of *tropical* genotypes, including the low-P-tolerant check Dular. Three SNPs at the 19,957,983rd, 19,959,498th, and 19,959,772th positions were deleterious (SIFT score less than 0.05) and resulted in change in isoleucine to threonine, glycine to serine and arginine to methionine, respectively. The SNP at the 19,959,772th position was also a splice variant. QTNGYPP2.2 spanned around 171 kb and had seventeen annotated loci, and two peak SNPs were found within *Os02g51570* (peptidyl-prolyl cis-trans isomerase) and *Os02g51670* (ethylene-responsive transcription factor). *Os02g51670* was considered a candidate gene and was also associated with PY. A nonsynonymous SNP (31,604,990th position) for *Os02g51610* was associated with GYPP (*p* value—4.18 × 10^−6^) and PY (*p* value—1.13 × 10^−6^). The QTN for GYPP on chromosome 5 (QTNGYPP5.1) spanned 151 kb and had four annotated loci; three peak SNPs each were present within two loci, *Os05g28180* (AMP deaminase) and *Os05g28190* (ranBP1 protein), respectively. Nine SNPs in *Os05g28200* (prenyltransferase) were associated with the peak SNP, and therefore, this was considered the candidate gene (Figure 2G). A haplotype analysis for the peak SNPs, splice variant (1,651,515th position) and associated SNPs from upstream and coding regions of the candidate gene identified three haplotypes, and it revealed that haplotype 1 had 2.9 g and 52 g higher GYPP and PY, respectively, than haplotype 3 (Figure 2H). The desirable haplotype was present in *indica*-type genotypes. QTNGYPP9.1 had twelve annotated loci, but SNPs were not detected within any annotated loci. Based on the LD block, nine SNPs within *Os09g23650* (FAM10 family protein), three in *Os09g23690* (UBX domain-containing protein), two in *Os09g23730* (HMG-Y-related protein) and three within *Os09g23740* (1-phosphatidylinositol-4-phosphate 5-kinase/zinc ion binding protein) were associated with the peak SNPs. The haplotype analysis for the seven nonsynonymous SNPs in the candidate gene *Os09g23650* identified five haplotypes with a difference of 2.7 g between haplotype 1 and haplotype 5 (Figure 2I–J). The desirable haplotype was present in 39% of *aus*-type genotypes, which include the low-P-tolerant check Dular. The SNP at the 14,051,302th position causes a change in aspartic acid to asparagine, which might exert a negative effect on GYPP. Apart from the above-mentioned haplotypes, other single-peak genic SNPs were also detected for GYPP, like *Os06g50360* (a pseudouridine synthase), *Os11g42230* (OsFBX430 F-box protein), and transporter proteins like *Os03g43720* and *Os07g47100* (Table 2).

To confirm the association observed for the grain yield per plant (GYPP), the GWAS for the plot yield (PY) was also performed. For PY, four QTNs were identified (details given in Supplementary Figure S5 and Table 1). The QTN for PY on chromosome 2 (QTNPY2.1) was 182 kb in length and had thirteen annotated loci; within this QTN, there were 96 significant SNPs, and a few were within the six annotated loci. *Os02g48110*, encoding a heat shock protein and having six nonsynonymous SNPs, was considered the candidate gene (Figure 2C). A haplotype analysis identified four haplotypes with an increase of 62 g of PY and 2 g of GYPP between haplotype1 and 4 (Supplementary Figure S5D,E). The SNP at the 2,94,55,392th position causes a change from arginine to histidine and might affect PY (sift-0.03). The desirable haplotype1 was present in all *indica*-type genotypes, including the tolerant check Swarna. Another locus within this region that had significant peak SNPs was *Os02g48100* (DEAD-box ATP-dependent RNA helicase). The second QTN on chromosome 2 for the plot yield (QTNPY2.2) was 106 kb in length and had nine annotated loci, and one peak SNP each was detected in loci *Os02g51550*, Os02g51570, *Os02g51610*, *Os02g51730*, *Os02g51740* and *Os02g51680* (trehalose-6-phosphate phosphatase). All of these genes were also detected in QTNGYPP2.2. In this QTN, *Os02g51670* (ethylene-responsive factor) had two SNPs and was considered a candidate gene (Supplementary Figure S5F–H). Since the locus did not have any nonsynonymous SNPs in our panel, a haplotype analysis was performed with all the other SNPs in exons and UTR. Four haplotypes were identified, and haplotype 1 had 1 g and 19 g higher GYPP and PY, respectively, than haplotype 4. The desirable haplotype in this locus predominantly occurred in the *aus*-type population. In QTNPY3.1, the peak SNP was not genic, but the next peak at 36,339,939 bp was associated with *Os03g64300* (thionin protein) (*p* value—2.18 × 10^−6^), which is a known plant defense protein (Supplementary Figure S5A). Three nonsynonymous SNPs present in this gene were used for haplotype analysis, and four haplotypes were identified, with haplotype 1 having a 1.3 g (GYPP) and 59 g (PY) yield advantage over haplotype 4 (Supplementary Figure S5I–K). The SNP at the 36,344,929th position causes a proline-to-leucine change with a minimum SIFT score (0) suggesting a negative effect on PY. The desirable haplotype with a positive effect on PY is present in 72% of the *aus*-type population, including the check IAC47. QTNPY8.1 spanned 125 kb in length and included 13 annotated loci, among which two significant peak SNPs were present in *Os08g43400* (kinesin motor domain-containing protein) (*p* value—2.31 × 10^−6^), and it is considered a candidate gene. Three other loci, namely *Os08g43370* (6-phosphogluconolactonase), *Os08g43380* (TBC domain-containing protein) and *Os08g43540* (peptidase), also exhibited a moderate association with the peak SNP. The haplotype analysis of the candidate gene *Os08g43400* with its three nonsynonymous SNPs identified four haplotypes, with haplotype 1 having a 13 g increase in PY compared to haplotype 4. The desirable haplotype was present in 72% of the *aus*-type population, including the low-*p*-tolerant checks Dular and Kasalath. Seven loci, viz. *Os01g57110*, *Os04g38530*, *Os05g25560*, *Os06g17290*, *Os07g27140*, *Os11g34110* and *Os12g05040*, with a single peak SNP near them, were also detected for PY (Table 2).

Two QTNs were detected for the trait tiller number (TN) on chromosomes four and eight (Table 1; Supplementary Figure S6). The QTNTN4.1 had three SNPs and spanned 23 kb in length. Based on the SNP likelihood LD block, methyltransferase (Os04g31000) protein-coding genes were identified as the candidate gene. A haplotype analysis for the gene showed three possible haplotypes based on the seven nonsynonymous SNPs within the gene and a peak SNP (18,532,574) located at the 3′ UTR region. The SNP at 18,522,640 bp had the lowest SIFT score and may cause a change in the protein conformation and affect the TN in rice plants. Haplotypes 1 and 2 showed significantly higher TN than haplotype3, and the desirable haplotype 1 was present in the tolerant checks Dular and Swarna. QTNTN8.1 spanned around 583 kb and had ten peak SNPs in them. Based on the LD block, the candidate gene was identified to be no apical meristem (Os08g42400) protein coding. Six haplotypes were identified for this gene, which includes two SNPs in 5′ UTR, two nonsynonymous SNPs in the second exon, one SNP in 3′ UTR and two peak SNPs in the downstream of the gene. A higher TN was observed in haplotype 1 (8.2 ± 0.6), followed by haplotype 2 (7.3 ± 0.5), and the other haplotypes showed a similar tiller number (6.5–6.8).

### 2.6. Genome-Wide Association Study and Haplotype Analysis for Phosphorus Utilization Efficiency and Related Traits

**Figure 3:**
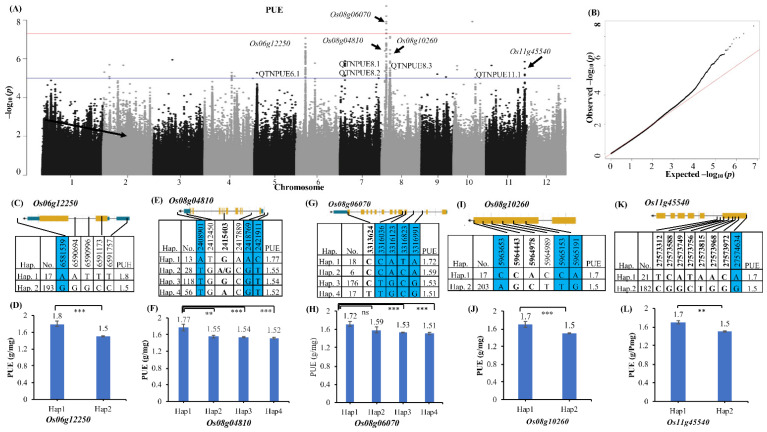
Identification of candidate genes associated with phosphorus utilization efficiency (PUE). (**A**,**B**) Manhattan plot and Q–Q plot of PY, GYPP and PUE with EMMAX model showing QTNs and associated candidate genes (highlighted with arrows). Horizontal lines in the Manhattan plots indicate the genome-wide thresholds -log *P* values of 5 (blue) and 7.5 (red). The dashed (red) line in Q–Q plot represents significance threshold, whereas black dots represent observed values. Gene structure of candidate genes—(**C**) *Os06g12250*, (**E**) *Os08g04810*, (**G**) Os08g06070, (**I**) *Os08g10260*, and (**K**) *Os11g45540* with a haplotype analysis of peak SNPs. Orange, blue and white colors represent exons, UTR and introns, respectively. Nonsynonymous SNPs are in bold; the blue-colored columns represent peak SNP. The average value for a particular haplotype (hap.) for PUE, along with the number of genotypes (no.), is indicated. (**D**,**F**,**H**,**J**,**L**) Phenotypic variation among haplotypes for PUE with significant values (*t*-test) indicated as ** (*p* < 0.01) and *** (*p* < 0.001), respectively, for candidate genes.

Five significant QTNs were detected for the trait PUE, and a haplotype analysis for all the candidate genes was performed (Figure 3).

The QTN for PUE on chromosome six (QTNPUE6.1) had 59 SNPs in a span of 231 kb, with SNPs detected in three loci, *Os06g12250*, *Os06g12260* and *Os06g12280*. Based on a local LD plot, *Os06g12250* (sphingolipid C4-hydroxylase SUR2) (*p* value—8.55 × 10^−8^) is considered the candidate gene (Figure 3C). A haplotype analysis of this gene revealed that haplotype 1 exhibited an increase of 0.3 g/mg of PUE over haplotype2 (Figure 3D). Three QTNs were detected on chromosome eight, QTNPUE8.1 was 13 kb in length and had three significant SNPs that were associated with *Os08g04810* (Figure 3E,F). QTNPUE8.2 was 104 kb in length and had 58 significant SNPs detected in the region, which includes SNPs in *Os08g06090*, *Os08g06100*, *Os08g06180*, *Os08g06190* and *Os08g06070*. Based on a local LD block, *Os08g06070* (ELF7) (Figure 3G,H) was identified as the candidate gene. Here, haplotypes 1 and 2 had a higher PUE than the other two haplotypes. Four significant SNPs were present in chromosomes 8 and 11. QTNPUE8.1 had two peak SNPs directly within the locus *Os08g10260* (NBS-LRR) (Figure 3I,J). An LD block analysis around the peak SNPs of QTNPUE11.1 identified locus *Os11g45540* as the candidate gene. A haplotype analysis for the candidate genes of QTNPUE8.3 and QTNPUE11.1 revealed that haplotype 1 exhibited an increase in PUE of 0.3 g/mg compared to haplotype 2. All the desirable haplotypes for PUE identified in this study were present only in *indica*-type genotypes, and hence, these could be used as donors in breeding for low-*p* tolerance under acidic soil conditions.

Seven single SNPs were also detected for FGPP (Table 2). Two loci, *Os08g03260* and *Os08g37904*, code for zinc finger protein. *Os08g02996* and *Os07g43040* code for a receptor-like kinase and a heavy metal-associated domain-containing protein, respectively. Three SNPs in chromosome 1 in genes coding for FAD-dependent oxidoreductase, cupin and methyltransferase were also identified (Table 2). SNPs were also detected for all the other traits studied, but an annotated potential candidate gene could not be identified, and hence, they are not discussed in this paper. Peak SNPs present in introns of 16 annotated loci for the traits GYPP, DM, TN, FGPP and PUE were also identified (Supplementary Table S7).

## 3. Discussion

The lowland rice fields of CPGS-AS, Meghalaya, have not received any inorganic P fertilizer input for at least the past 10 years, and the soil is acidic [9,10,17]. Due to the low pH in acidic soils, the available inorganic P is converted into non-available forms by binding with aluminum, iron and other metal oxides, leading to a reduction in the rice yield. Comparable low yields (less than 2 tonnes per hectare) are reported for the acidic soils of Malaysia [26] and Indonesia [27]. Therefore, identifying alleles, genes and loci associated with low-P tolerance in rice will help utilize them in the development of low-P-tolerant, high-yielding rice varieties.

Two hundred and thirty-four diverse rice genotypes (grouping into three *indx*, *ind2* and *aus* distinct subgroups) belonging to 3K RDP were grown in the low-input acidic soils for two consecutive seasons in an augmented block design with checks. Generally, a mixed-model analysis is used to find the best linear unbiased estimation (BLUE) of fixed effects or best linear unbiased predictions (BLUP) of random effects, and they are proven to be effective for analyzing phenotypic and SNP data [28]. In this study, BLUP values were calculated using data from two seasons, and there were significant variations for yield, P, and hydroponics-related traits, with high heritability for yield-related traits. The reported average yield per plant of genotypes grown in low-P soils was 12.9 [29] and 10 g [30], which was similar to the average grain yield per plant of our genotypes at 9.9 g in the combined analysis. The average phosphorous content (PC) and phosphorous utilization efficiency (PUE) obtained in this study were 0.8 mg and 1.5 g/mg, respectively, and it was similar to values obtained previously [31,32]. There was a decrease in the shoot weight because of P deficiency. Seventeen genotypes including SD had a positive relative shoot fresh weight (RSFW) in hydroponics, which indicates tolerance [6,10]. A positive correlation between PUE and biomass is important for efficiency [31]. Phosphorous utilization efficiency (PUE) and relative shoot fresh weight (RSFW) had a positive correlation with most of the yield and its related traits. Based on observations in the study, ARC10145 and Selhi were the extremely low-P-tolerant and susceptible genotypes, respectively. Ward’s clustering identified three clusters, and genotypes of cluster II can be directly used as parents in breeding for high yield under low-P acidic soils.

### 3.1. Peak SNPs Identified in Genes Involved in Abiotic Stress Tolerance in 1.8 Mb Region of Chromosome 2

The short arm of chromosome 2 carries many QTLs, including qDTY2.2 [24] and qGY2.4 [33], associated with grain yield under drought stress. Our previous study using a biparental population [10] also identified a much smaller region of 1.8 Mb lying within these QTLs that was associated with the grain yield in acidic soils. In the current study, polymorphic SNPs identified in this region for 235 diverse genotypes were analyzed for their association with yield and low-P tolerance-related traits in acidic soils, and twenty-five annotated loci were identified. *Os02g09840* (*OsPP2A*) was identified as the candidate gene governing the grain yield per plant (GYPP) and plot yield (PY) in this 1.8 Mb region. The *OsPP2A* gene is involved in various regulating signaling pathways, including growth, biotic and abiotic stresses [34]. In rice, the members of this gene family are involved in panicle, seed developmental stages and drought, salinity and heat stress [35]. In maize, a similar gene (*ZmPP2AA1*) is known to be involved in root development and auxin signaling under low-P responses [36]. In the current study also, a deleterious SNP variant (5,073,629th position) in this gene caused a significant change in both PY and GYPP. The nearby gene *Os02g09830* (*OsZIP*16) is also known to be positively regulated under drought stress [37]. GWAS identified a cinnamoyl CoA reductase gene (*Os02g08420*) for PUE within this region. This is a lignin biosynthesis gene involved in the regulation of the phenylalanine metabolic pathway, and it is reported to be upregulated in copper stress [38], cell wall-related stress [39] in rice and low-P stress in *Pinus massoniana* [40].

### 3.2. Candidate Genes for Higher Yield Under Low-P Acidic Soils Identified Through GWAS

Among five QTNs detected for GYPP, the most significant was QTN11.1. The candidate gene *Os11g34110* (*OsHGSNAT*) was also detected for the traits PY and DM. The closest gene, *Os11g34120* (exportin 1 protein), is required for various developmental stages, and it regulates abiotic stress tolerance. *OsHGSNAT* is a transmembrane enzyme known to upregulate under low-P starvation, and it causes changes in the cell walls of rice shoots [41]. A GWAS study identified *HGSNAT* as a putative gene for an increase in the grain yield of wheat grown under stress [42]. This gene was also reported to be upregulated and show decreased methylation under cadmium stress in tobacco [43]. *HGSNAT* is also known to play a role in sugar metabolism and thereby cause tomato ripening [44]. An increase in the sucrose and starch content of rice leaves subjected to P deficiency has been reported in many studies [45]. Three SNPs in this gene might lead to a change in the protein conformation and function, which might cause cell-wall remodeling and increase the carbohydrate metabolism and thereby increase the grain yield of rice. This needs further validation. A chloroplast synthase gene (*OsCHLG*) underlies the QTNGYPP5.1 detected in this study. Alternate wetting and drying are known to alleviate low-P stress in rice, and under these conditions, the *OsCHLG* gene was upregulated in the flag leaf of rice [46]. It is downregulated under alkaline stress [47]. Another significant gene in this QTN, *Os05g28180*, a nucleotide metabolism-related gene, is downregulated under P deficiency [48] and upregulated under potassium deficiency [49]. A thioredoxin protein, *Os09g23650*, underlies QTNGYPP9.1 which is involved in modulating the redox status of phosphate over accumulator (*OsPHO*) gene under P deprivation. SNPs detected in single-candidate genes are also reported for several stresses. For example, *Os06g50360* was upregulated under salt stress [50] and cold stress [51], and nearby *Os06g50380* is a candidate gene for aluminum tolerance [52]. *Os02g51670* (*OsDREB2B*), the candidate gene underlying QTN for GYPP on chromosome 2 (QTNGYPP2.2), is reported to play a negative role in rice growth and development [53].

Loci and candidate genes detected for PY were mostly reported to be expressed in P-related and other stresses, and they indirectly affected the grain yield. Among the four QTNs detected for PY, the most significant was on chromosome 2. A heat shock protein-HSP70 (*Os02g48110*) underlying the QTNPY2.1 is expressed under heat and biotic stress conditions [54], and various classes of HSP are expressed under low-P stress as well [55]. Three nonsynonymous SNPs in this HSP included a deleterious variant, and they can change the protein conformation and enhance low-P stress tolerance. Two QTNs on chromosome 2, namely QTNGYPP2.2 and QTNPY2.2, overlap a previously identified QTL in chromosome 2 governing TN, DM, FGPP and P uptake under low-P stress in rice [56]. Apart from the candidate gene *Os02g51670* (*OsDREB2B*) for QTNPY2.2 and the nearby gene, *Os02g51680* (*OsTPP7*) too, is reported to be involved in starch mobilization in anaerobic germination tolerance [57]. The genes in the QTNPY2.2 govern tolerance/responses to many biotic and abiotic stresses, and therefore, identified ‘desirable’ haplotypic combinations can be utilized in future breeding programs addressing climate change. QTNPY3.1 harbored the thionin protein as a candidate gene. Three different thionin proteins were previously reported to be upregulated in rice shoots under low-P starvation [58]. QTNPY8.1 had a peak SNP in *Os08g43400* (kinesin motor domain), which is involved in cellulose deposition and microfibril assembly in rice shoots. Other significant genes in this QTN, like *Os08g43370* and *Os08g43380*, affect the shoot biomass under salt stress [59]. This suggests that genes in this QTN increase the shoot biomass under stress and could thereby increase the grain yield. Single SNPs detected for PY were also previously identified for various stresses. A mutation in the *Os01g57110* gene resulted in alkaline stress tolerance [60], *Os04g38530* was downregulated under P stress in rice leaves [61] and *Os12g05040* is known to be involved in iron stress [62].

One of the candidate genes identified for TN present in QTNTN4.1 is an ethyltransferase (*Os04g31000*), previously detected in a QTN for root thickness [61]. But the role of this gene in increasing the tiller number has yet to be identified. The other identified gene (*Os08g42400*/*Os08g0535800*) is a member of NAC transcription family reported to be involved in various plant physiological processes like tillering and stress response [63]. Close to the methyltransferase gene is a nitrate-induced protein (Os04g31030), which is significantly connected with *Os08g42400* and other genes, as detected via the clustering coefficient [64], suggesting a gene co-expression pattern. Hence, both the identified candidate genes might be activated simultaneously as a network and increase the tiller number, as well as the panicle number (the QTNs identified for the tiller number in this study were also observed for the panicle number.

### 3.3. Candidate Genes Identified for PUE and Other Related Traits

Five QTNs for PUE were identified in this study were novel, with several underlying genes reported to be expressed under various biotic and abiotic stresses. The sphingolipid C4-hydroxylase (*OsSUR2*) gene identified in QTNPUE6.1 was highly expressed under heat stress [65], and it is involved in rice blast tolerance and might positively regulate abiotic tolerance [66]. The mutation in *SUR2* genes affected glucosinolate biosynthesis and increased the auxin accumulation, thereby producing more adventitious roots in *Arabidopsis* [67]. Similar mechanisms might increase the root growth in rice under P deficiency. The candidate gene serine esterase/hydrolase (*OsSH*) (*Os08g04810*) in QTNPUE8.1 is involved in lipid metabolism, plant development and defense responses [68]. Serine esterase gene is known for lipid mobilization in growing rice seedlings [69]. An *ELF7*/*PAF1* gene identified in QTNPUE8.2 was previously identified for BLB resistance in a GWAS study [70]. A mutation in the *PAF1* gene in *Arabidopsis* increased arsenic tolerance [71]. Phosphorus uptake suppresses the arsenic uptake in rice [72], and hence, allelic variants identified in this gene might eventually increase the P uptake and utilization efficiency. Two disease resistance genes were identified in QTNPUE8.3 and QTNPUE11.1. The *NBS-LRR* genes are known to play a role in rice blast disease, and the receptor-like kinase gene, *OsRLCK352* (*Os11g45540*) is reported for bacterial leaf blight [73]. The role of these disease-resistance genes in governing P utilization, if any, needs to be evaluated.

Almost all the detected SNPs for FGPP were previously reported for different stages of seed development. *Os01g59490* (FAD-dependent oxidoreductase) is expressed in four out of five stages of seed development [74], and *Os08g02996* is essential for stigma and ovary development [75]. Methyltransferase and cupin genes are also essential in the early stages of seed development [76,77]. Zinc fingers and *C2H2* type zinc fingers are involved in seed development and abiotic stress tolerance [78]. *Os07g43040* (heavy metal-associated domain-containing protein) is known to increase grain zinc and iron [79]. These genes were specially activated under low-P stress conditions and increased filled grains per panicle. The identified haplotypes and the responsible candidate genes though involved in different pathways can play a crucial role in combating the low-P stress and other related stress in acidic soils.

## 4. Materials and Methods

### 4.1. Planting Material

A subset of 3K RDP, comprising 234 genotypes, was obtained from IIRR (Indian Institute of Rice Research, Hyderabad) (Supplementary Table S1) and grown at the experimental farm of College of Post-Graduate Studies in Agricultural Sciences (CPGS-AS), Meghalaya, India, for two consecutive seasons (kharif, 2020 and kharif 2021) using an augmented randomized block design with twenty-four plants per genotype. In kharif, 2020, sufficiently available 208 genotypes (26 genotypes were not included, as sufficient data were not obtained) were grown in ten blocks with six checks, namely Sahbhagidhan (SD), Kasalath (PSTOL^+^), Shasharang (PSTOL^−^), IR1552—susceptible to low P [25], CAUS105 (PSTOL^+^)—an advanced breeding line of CPGS-AS, and CGZRI—a low-P susceptible variety (unpublished data). Checks were replicated and maintained in the middle of all blocks, whereas genotypes were planted only once. These six checks are the standard checks used in the rice breeding program at CPGS-AS. In kharif 2021, all 234 genotypes were grown in the same field with ten blocks and twelve checks (six additional checks included mega varieties, namely Chandrahasini, CR Dhan 40, DRR Dhan 48, Protazin, Swarna and Zinco rice ms). Checks like Swarna (PSTOL^+^) and CR Dhan 40 (PSTOL^−^) are reported to be tolerant of low P [6]. However, the status of the remaining four checks with respect to low P was unknown.

### 4.2. Phenotyping for Agro-Morphological Traits and Pi Estimation

Data were taken from ten randomly selected plants within each plot and then averaged. Observations for ten yield-related parameters like the tiller number (TN), panicle length (PL) (cm), spikelets per panicle (SPP), filled grains per panicle (FGPP), spikelet fertility percentage (SF), biological yield (BY) (grams (g)), grain yield per plant (GYPP) (g), dry matter (DM: aboveground plant without panicles at harvest, dried and weighed in g), plot yield (PY: weight of threshed grains from 24 plants per plot in g) and harvest index (HI: [YPP/BY] × 100) were recorded.

Flag leaves of three plants per genotype were collected at harvest, oven-dried, pooled and weighed. A total of 0.5 g finely chopped shoots were digested using 5 mL of diacid (3 nitric acid: 1 perchloric acid). The shoot/flag leaf P concentration (PC: soluble Pi concentration in mg/g) was estimated using the phosphovanadate method [80] for both seasons, 2020 and 2021. From the PC, the phosphorous utilization efficiency (PUE) at harvest was calculated as 1/PC [81], which is defined as the dry matter produced per unit of P accumulated in shoot tissue [82].

### 4.3. Evaluation for Low-P Tolerance Under Hydroponics Condition

All the 234 genotypes and twelve checks described previously were screened for low-P tolerance in a hydroponic experiment using a completely randomized design. In brief terms, initially, the seeds were germinated on germination paper for six days and then transferred to netted floaters held in individual cups with around 300 mL of Yoshida nutrient medium (pH 4.5–5) and two replications in both the control and the treatment, with five plants per replication. For the first seven days, both the control and treatment groups exhibited an optimum concentration of nutrient solution with pH 5. After seven days, the treatment cups were filled with nutrient solution with low P by reducing NaH_2_PO_4_.2H_2_O to 0.015 mM, whereas the control had optimum nutrients with NaH_2_PO_4_.2H_2_O at 0.28 mM. Both the control and treatment cups were refilled with a freshly made nutrient solution every fifth day.

After 28–30 days, observations were recorded concerning traits like root length (RL: length from base of stem to tip of the longest root in cm), shoot fresh weight (SFW: fresh shoots were removed from roots and weighed in grams), root fresh weight (RFW: weighed in grams), total fresh weight (TFW: SFW + RFW) and total dry weight (TDW: plants were dried at 32 °C in greenhouse for 7 days and weighed in g) (Supplementary Table S1). The observations from five plants were averaged for each replication, and relative traits were calculated (relative trait = ((treatment − control)/control) × 100) [83]. The relative root length (RRL), relative shoot fresh weight (RSFW), relative root fresh weight (RRFW) and relative total fresh weight (RTFW) and relative total dry weight (RTDW) were calculated. Genotypes lacking sufficient plants were excluded from the analysis.

### 4.4. Statistical Analysis of Phenotypic Data

In this study, mixed linear models were used to obtain the best linear unbiased prediction (BLUP) values using the *lme4* [84] package in RStudio. Combined season analysis was performed by considering genotype (G), block (B), environment (E), and G × E (interaction) as random effects, and BLUP values were retrieved for all genotypes. These values were further used for GWAS, correlation and cluster analysis. The variance components obtained from the mixed-model analysis were used to calculate heritability (h^2^ in a broad sense) as h^2^ = Vg/(Vg + Vg_x_e + Ve), where ‘Vg’, ‘Vg_x_e’ and ‘Ve’ are variances of genotype, genotype x environment and error variances. The model fitting and estimations were used as mentioned by You et al. [85]. The frequency distribution with a standard error (Student’s ‘t-test’-one tailed, equal variance) was accomplished using MS Excel. The hydroponics data were analyzed for a two-way ANOVA with replications using the data analysis tool pack in MS Excel, where treatments (control and treatment) and genotypes were considered as factors. The least significant difference was calculated as LSD = t × $\sqrt{2\;EMS/r}$, where ‘t’ is the table ‘t’ value at the 0.05, 0.01 and 0.001 levels, ‘EMS’ is the error mean sum of the square of the trait and ‘r’ is the number of replications. LSD values were used to claim significance for hydroponics data. The Euclidean distance-based wards method of clustering genotypes for the selected traits was plotted using science and research online plot (SRplot, 2023) [86], a free R code-based platform for data analysis and visualization. A correlation analysis was performed in R studio using the *corrplot* package [87] and SRplot (2023) [86].

### 4.5. Quality Control, Threshold Identification and Association Study

The 29 million SNPs available in the Rice SNP-Seek database (2023) [14] were downloaded, and quality control (QC) was performed in the PLINK v2 software [88]. QC was performed for the SNPs of these 236 genotypes (234 test genotypes + 2 checks (Kasalath and Swarna)) with a missing rate of 10%, a minor allele frequency of 5% and a missing genotypes rate of 5%. After the QC, there were 3,443,005 polymorphic SNPs for 235 genotypes (the genotype ‘puttige’ was removed due to missing data). These 235 genotypes were subjected to single locus GWAS based on a linear mixed model in the EMMA eXpedited (EMMAX) software (version emmax-beta-07Mar2010) [89] using kinship matrix and PCA (principal component analysis) components. A kinship BN (Balding–Nichols) matrix was constructed using the EMMAX software version emmax-beta, and PCA analysis was performed in the PLINK software version 1.07 (http://pngu.mgh.harvard.edu/purcell/plink/). The PCA plot was constructed using eigenvector values in an XY scatter chart, and eigenvalues were plotted using a line chart in MS Excel, respectively. Based on the eigenvalues screen plot, first, three components were used as covariates (Q matrix) to account for the population structure (Supplementary Figure S1). The effective number of independent markers (N) was identified using the command ‘indep-pairwise 50 10 0.1’ in the PLINK software version 1.07, which identified 107,444 independent marker positions from which the *p* value was calculated as (1/N) 9.3 ×10^−6^. Thus, a threshold of >5 was set in the Manhattan plots to select for significantly associated SNPs/QTNs [90,91]. The Manhattan plots and their corresponding Q–Q plots were plotted using the *qqman* library in R studio (version 4.1.0). A heatmap of the kinship matrix was plotted using the *GAPIT* package (version 3) [92] in R studio. The population structure was identified in the ADMIXTURE software version 1.3.0 (https://dalexander.github.io/admixture/index.html) [93], and the Q matrix was plotted using a 100% stacked column chart in MS Excel 365.

### 4.6. Linkage Disequilibrium (LD) Decay, LD Plot, Gene Identification and Haplotype Analysis

LD decay is known to be between 100 and 300 kb in rice, and it varies between chromosomes and subpopulation types [90]. Hence, a minimum, i.e., 100 kb upstream and downstream of the peak SNPs, was searched for candidate gene prediction. The LD block around the peak SNP was plotted using the ‘SNP likelihood LD heatmap’ function in an SR plot. A QTN/QTL covers all the SNPs located within an LD region, and the SNP with the smallest *P* value was considered the peak SNP. The loci carrying the peak SNP were considered the candidate gene. The locus ID (RAP ID, MSU ID) and its annotations for all chromosomes were taken from the Rice Genome Annotation Project database (2023) [94]. Loci that were annotated as expressed, unknown, hypothetical and retrotransposon protein were ignored. The haplotype analysis was performed with peak SNPs and associated SNPs in the exon; 5′ UTR (untranslated region), 3′ UTR, 2 kb upstream and 1 kb downstream of an annotated locus were also utilized to find haplotypes [90]. The loci structure image was taken from the ‘JBrowser’ option available in the Rice SNP-Seek database (2023) [14]. All the details of the SNPs and their SIFT (Sorting Intolerant From Tolerant) scores were obtained from the Rice SNP-Seek (2023), Gramene (2023) [95] and RiceVarMap2 (2023) [96] databases. The SNP information available for checks like Swarna and previously known low-P-tolerant checks, including Kasalath, Dular and IAC47 [10], were also analyzed to find their corresponding haplotypes.

### 4.7. Association Study for a 1.847 Mb Region on Chromosome 2

The region from 4,073,883 bp to 5,967,668 bp, spanning a length of 1.847 Mb on chromosome 2 [10], was analyzed separately to find peak SNPs and associated loci. The SNP retrieval, QC and association study using EMMAX were performed as mentioned in the previous section. A total of 16,090 SNPs was used for analysis, and based on identified independent markers, the threshold was set to be <0.0023.

## 5. Conclusions

The candidate genes in the identified yield QTNs were previously reported in studies for low-P tolerance. Genes involved in carbohydrate metabolism, cell wall modification, heat, salinity, drought, BLB, leaf blast and related stress genes were detected for the traits grain yield per plant, plot yield and phosphorus utilization efficiency. The haplotypic combinations for the important candidate genes like *OsHGSNAT*, *OsCHLG*, *OsERTF*, *OsHSP70*, *OsSUR2*, *OsSH* and *OsPAF1* were shown to significantly increase yield and PUE, and they could be utilized in future breeding programs.

## Figures and Tables

**Table 1:** List of QTNs detected through GWAS analysis for the traits GYPP, PY, TN and PUE.

Trait	QTN	Physical Position	Peak SNP	*p* Value	No.	Associated Loci	Annotation
GYPP	QTNGYPP2.2	31583066–31756679	31583481	4.18 × 10^−6^	18	LOC_Os02g51670/ Os02g0752800	Ethylene-responsive transcription factor/dehydration-responsive element-binding protein 2B
	QTNGYPP5.1	16365441–16514559	16376571	9.79 × 10^−7^	50	LOC_Os05g28200/ Os05g0349700	Prenyltransferase/chloroplast synthase
	QTNGYPP9.1	13899180–14143315	13899180	2.33 × 10^−6^	4	LOC_Os09g23650/ Os09g0401200	*FAM10* family protein/tetraticopeptide domain-containing thioredoxin
	QTNGYPP11.1	19883002–19959555	19948634	9.91 × 10^−7^	40	LOC_Os11g34110/ Os11g0543500	Heparan-alpha-glucosaminide N-acetyltransferase
PY	QTNPY2.1	29338187–29528492	29402867	7.43 × 10^−7^	96	LOC_Os02g48110/ Os02g0710900	*DnaK* family protein/ heat shock protein (*Hsp70*)
	QTNPY2.2	31574786–31681012	31629041	1.13 × 10^−6^	91	LOC_Os02g51670/ Os02g0752800	Ethylene-responsive transcription factor/dehydration-responsive element-binding protein 2B (*DREB2B*)
	QTNPY3.1	36339939–36362784	36339939	2.18 × 10^−6^	3	Loc_Os03g64300/ Os03g0860900	*THION30*—plant thionin family protein precursor/ *WD40* repeat-like protein
	QTNPY8.1	27334831–27459981	27448010	2.31 × 10^−6^	3	LOC_Os08g43400/ Os08g0547500	Kinesin motor domain-containing protein
PUE	QTNPUE6.1	6456833–6687984	6581539	8.55 × 10^−8^	59	LOC_Os06g12250/Os06g0226950	Sphingolipid C4-hydroxylase *SUR2*/Fatty acid hydroxylase
	QTNPU8.1	2408901–2421911	2408901	3.87 × 10^−7^	3	LOC_Os08g04810/Os08g0143700	Serine esterase/hydrolase
	QTNPU8.2	3312360–3416391	3324300	1.88 × 10^−9^	58	LOC_Os08g06070/Os08g0157100	*ELF7*/*Paf1* domain
	QTNPU8.3	5958930–5965191	5965191	4.92 × 10^−6^	4	LOC_Os08g10260/Os08g0202400	*NBS-LRR*/disease resistance protein
	QTNPUE11.1	27491496–27574635	27491496	1.4 × 10^−6^	4	LOC_Os11g45540/Os11g0681400	*TKL_IRAK_DUF26*-lh.11—*DUF26* kinases
TN	QTNTN4.1	18509344–18532574	18509683	2.57 × 10^−6^	3	LOC_Os04g31000/Os04g0379300	Methyltransferase domain-containing protein
	QTNTN8.1	26190355–26773599	26773599	5.75 × 10^−6^	10	LOC_Os08g42400/ Os08g0535800	No apical meristem protein (*NAM*)

No.—Number of SNPs within the QTN region.

**Table 2:** List of individual peak SNPs and putative candidate gene identified from GWAS analysis.

Trait	Chr.	SNP Position	*p* Value	Candidate Gene	Annotation
GYPP	3	24425242	5.83 × 10^−6^	Os03g43720	Transporter family protein
	6	30475205	9.64 × 10^−6^	Os06g50360	Pseudouridine synthase family protein
	7	28158176	3.58 × 10^−6^	Os07g47100	Transporter, monovalent cation: proton antiporter−2 family
	11	25436658	9.28 × 10^−6^	Os11g42230	*OsFBX430*—F-box domain-containing protein
PY	1	33040531	2.61 × 10^−7^	Os01g57110	*SNF2* family N-terminal protein
	4	22897241	8.88 × 10^−6^	Os04g38530	Aldose 1-epimerase
	5	14854900	2.77 × 10^−8^	Os05g25560	Glycosyl hydrolase family 10 protein
	6	10036143	7.20 × 10^−6^	Os06g17290	Phosphatidylinositol 3- and 4-kinase protein
	7	15821103	3.41 × 10^−6^	Os07g27140	AT hook motif family protein
	11	19958448	5.85 × 10^−6^	Os11g34110	Heparan-alpha-glucosaminide Nacetyltransferase
	12	2200441	5.33 × 10^−6^	Os12g05040	Heavy-metal-associated protein
DM	11	19958448	2.63 × 10^−6^	Os11g34110	Heparan-alpha-glucosaminide Nacetyltransferase
FGPP	1	34407559	8.37 × 10^−7^	Os01g59490	FAD-dependent oxidoreductase domain-containing protein
	1	34419588	4.10 × 10^−6^	Os01g59520	Cupin, RmlC-type
	1	36373428	8.65 × 10^−6^	Os01g62800	Methyltransferase
	7	25792255	7.45 × 10^−6^	Os07g43040	Heavy metal-associated protein
	8	1311795	1.03 × 10^−6^	Os08g02996	Receptor-like kinase
	8	1512058	2.99 × 10^−6^	Os08g03260	Zinc finger family
	8	24009532	4.31 × 10^−6^	Os08g37904	*ZOS8-08—C2H2* zinc finger
PUE	2	4536291	2.02 × 10^−6^	Os02g08420	cinnamoyl CoA reductase

Chr.—Chromosome number; GYPP—grain yield per plant; PY—plot yield; DM—dry matter; FGPP—filled grains per panicle; PUE—phosphorus utilization efficiency.

## Data Availability

The phenotypic data used in this study are presented in additional file 1: Supplementary Tables S4 and S5. The SNP genotypic data used were downloaded from the Rice SNP-Seek database.

## Supplementary Materials

The following supporting information can be downloaded at: https://www.mdpi.com/article/10.3390/ijms252111673/s1.
