# Supplementary material for: Genome-Wide Association-Based Identification of Alleles, Genes and Haplotypes Influencing Yield in Rice (Oryza sativa L.) Under Low-Phosphorus Acidic Lowland Soils
Source: Int J Mol Sci. 2024 Oct 30;25(21):11673. doi: 10.3390/ijms252111673 (PMC11546970; doi:10.3390/ijms252111673)
Supplement: Supplementary file 1 [file ijms-25-11673-s001.zip › SupplementaryfiguresS1-S6.pdf]

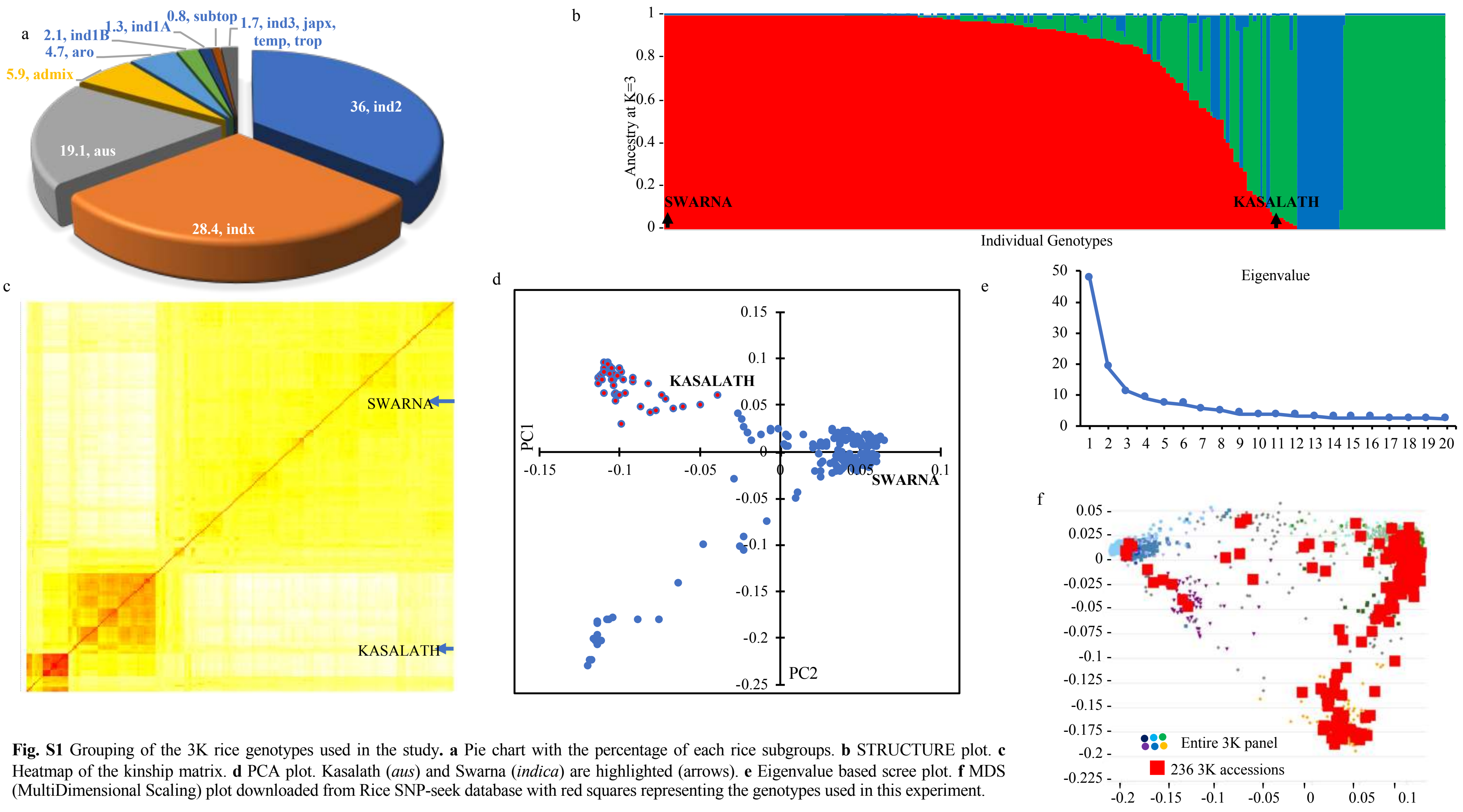


**Fig. S1** Grouping of the 3K rice genotypes used in the study**. a** Pie chart with the percentage of each rice subgroups. **b** STRUCTURE plot. **c** Heatmap of the kinship matrix. **d** PCA plot. Kasalath (*aus*) and Swarna (*indica*) are highlighted (arrows). **e** Eigenvalue based scree plot. **f** MDS (MultiDimensional Scaling) plot downloaded from Rice SNP-seek database with red squares representing the genotypes used in this experiment.

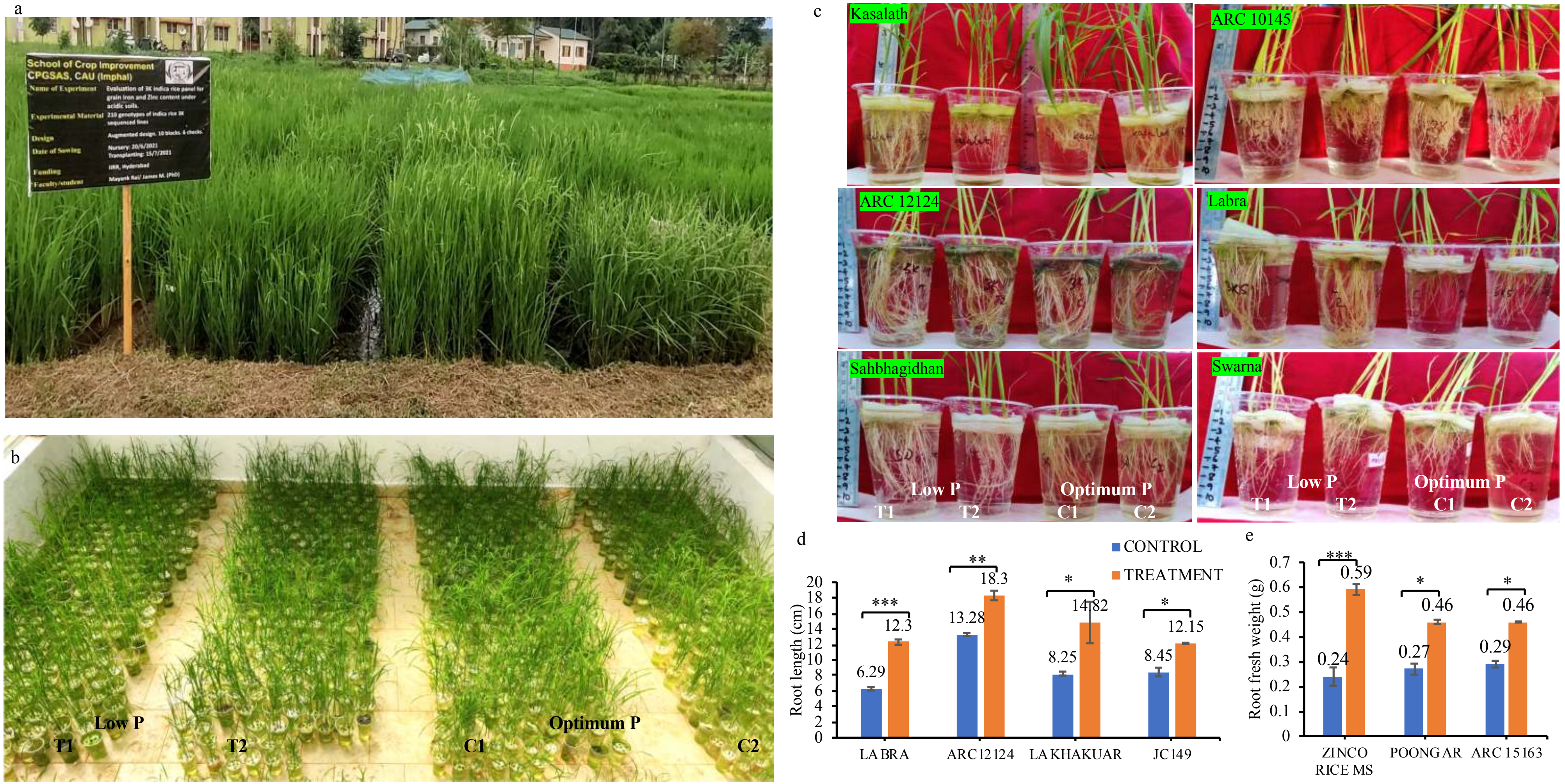


**Fig. S2** Representative pictures at different stages of the experiment- **a** rice field with 3K genotypes showing variation in kharif 2021. **b** hydroponics experiment in greenhouse. **c** representative picture of checks and few genotypes showing difference in root length and biomass. **d-e** Difference in the phenotypic means for traits root length (RL) and root fresh weight (RFW) in hydroponics experiment. Asterisks * ($P < 0.05$), ** ($P < 0.01$) and *** ($P < 0.001$) on the top of bars indicate significant difference in phenotypic values between control and treatment.

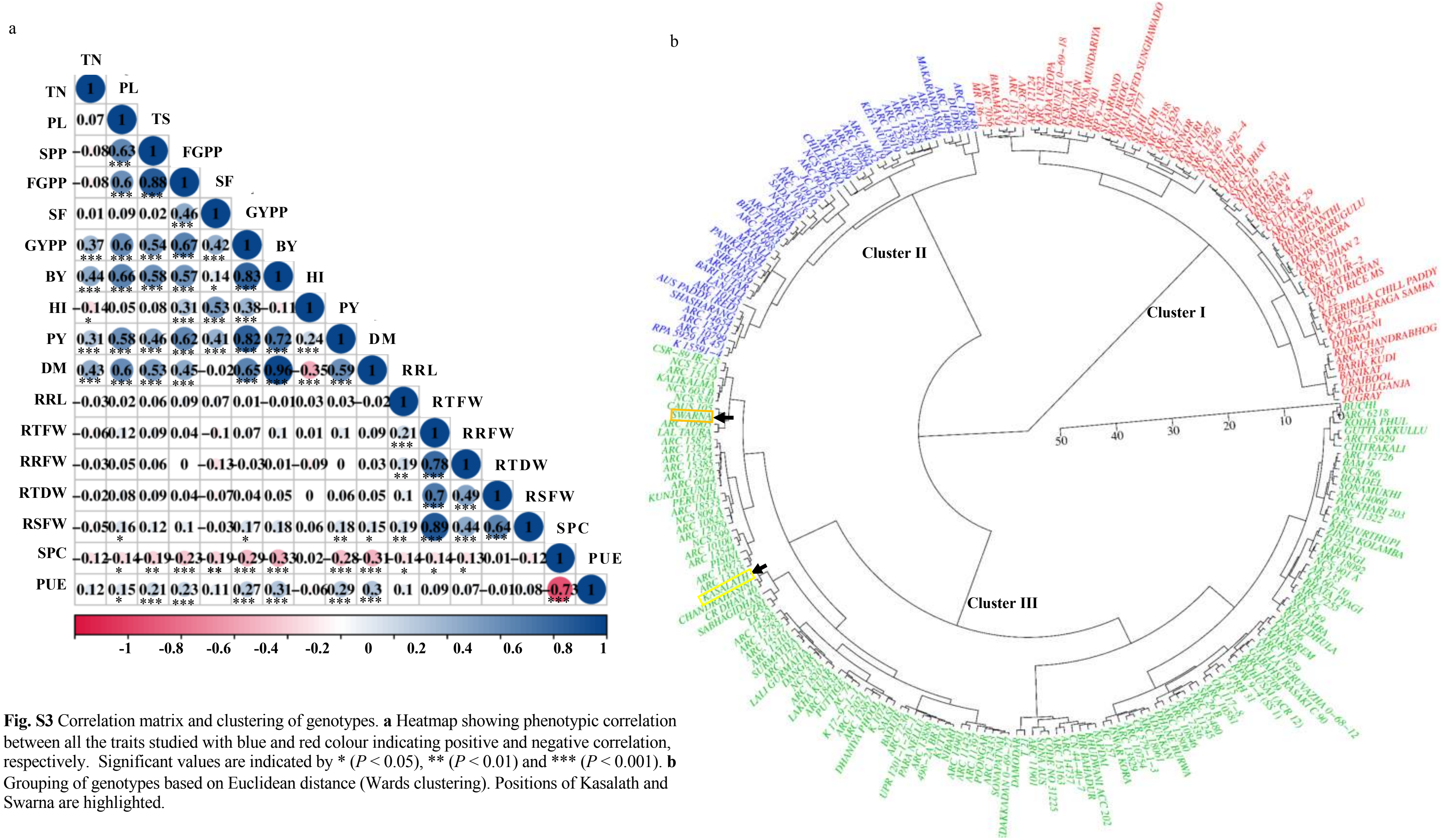


**Fig. S3** Correlation matrix and clustering of genotypes. **a** Heatmap showing phenotypic correlation between all the traits studied with blue and red colour indicating positive and negative correlation, respectively. Significant values are indicated by * ($P < 0.05$), ** ($P < 0.01$) and *** ($P < 0.001$). **b** Grouping of genotypes based on Euclidean distance (Wards clustering). Positions of Kasalath and Swarna are highlighted.

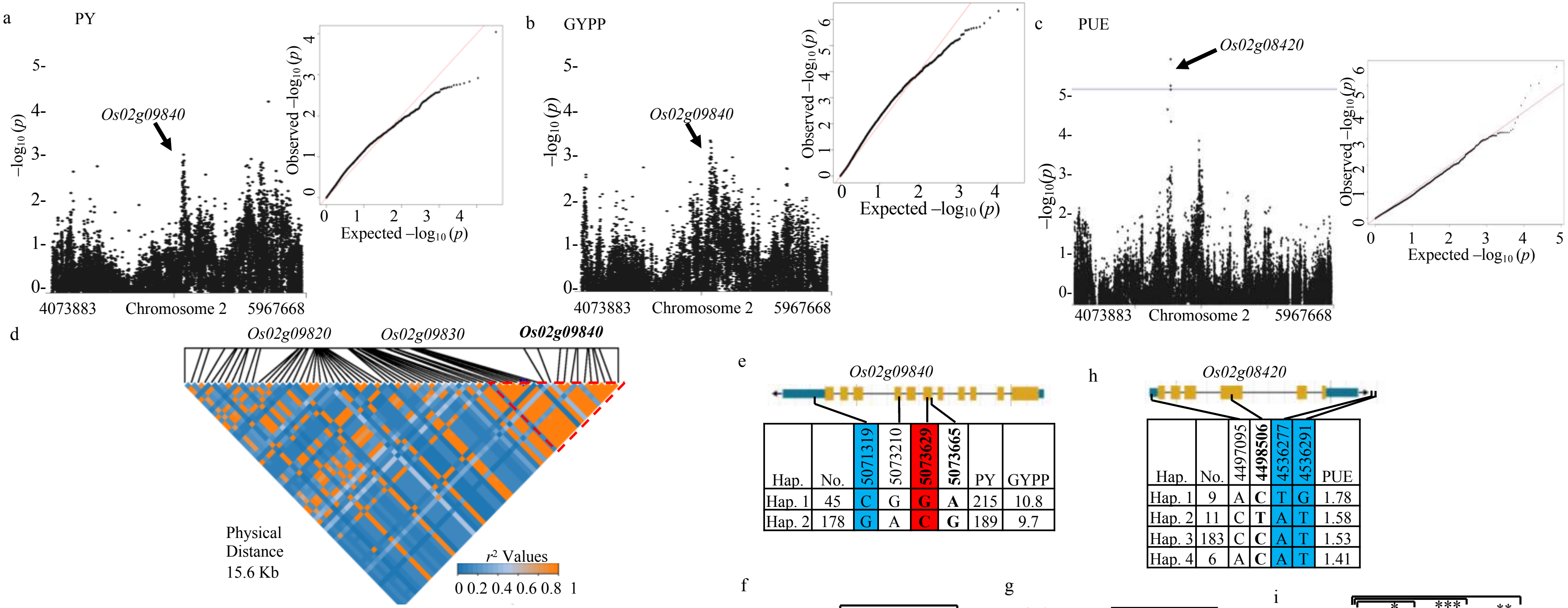

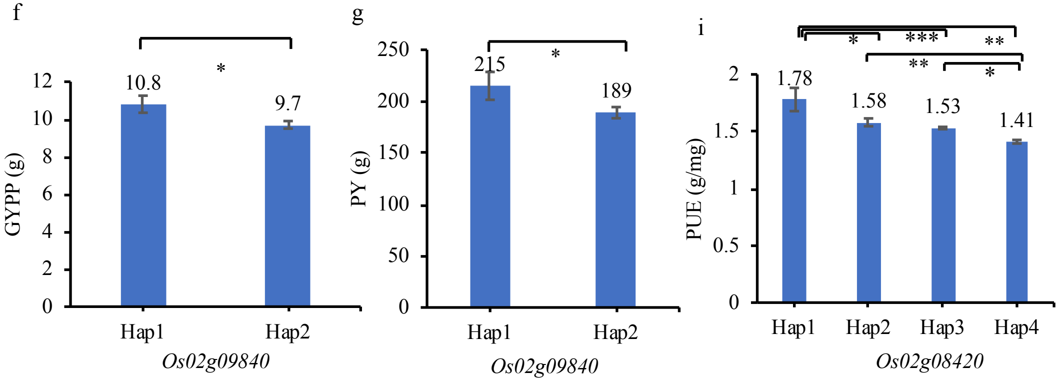


**Fig. S4** Identification of candidate genes associated with PY, GYPP and PUE in the previously reported region on chromosome 2. **a-c** Manhattan plot and Q-Q plot of PY, GYPP and PUE with EMMAX model showing QTNs and associated candidate genes (highlighted with arrows). **d** Zoom in SNP likelihood LD (Linkage Disequilibrium) heatmap showing the position of candidate gene *Os02g09840* within dotted red triangle. Gene structure of candidate genes- **c** *Os02g09840*; **h** *Os02g08420* with haplotype analysis of peak SNPs. Orange, blue and white colour represent exons, UTR and introns, respectively. Nonsynonymous SNPs are in bold; blue and red-coloured columns represent peak, and deleterious SNP (SIFT score < 0.05), respectively. Average value for a particular haplotype (hap.) for GYPP, PY and PUE along with number of genotypes (no.) is indicated. **f-g; i** Phenotypic variation among haplotypes for GYPP, PY and PUE with significant values (*t* test) indicated by * ($P < 0.05$), ** ($P < 0.01$) and *** ($P < 0.001$), respectively for candidate genes.

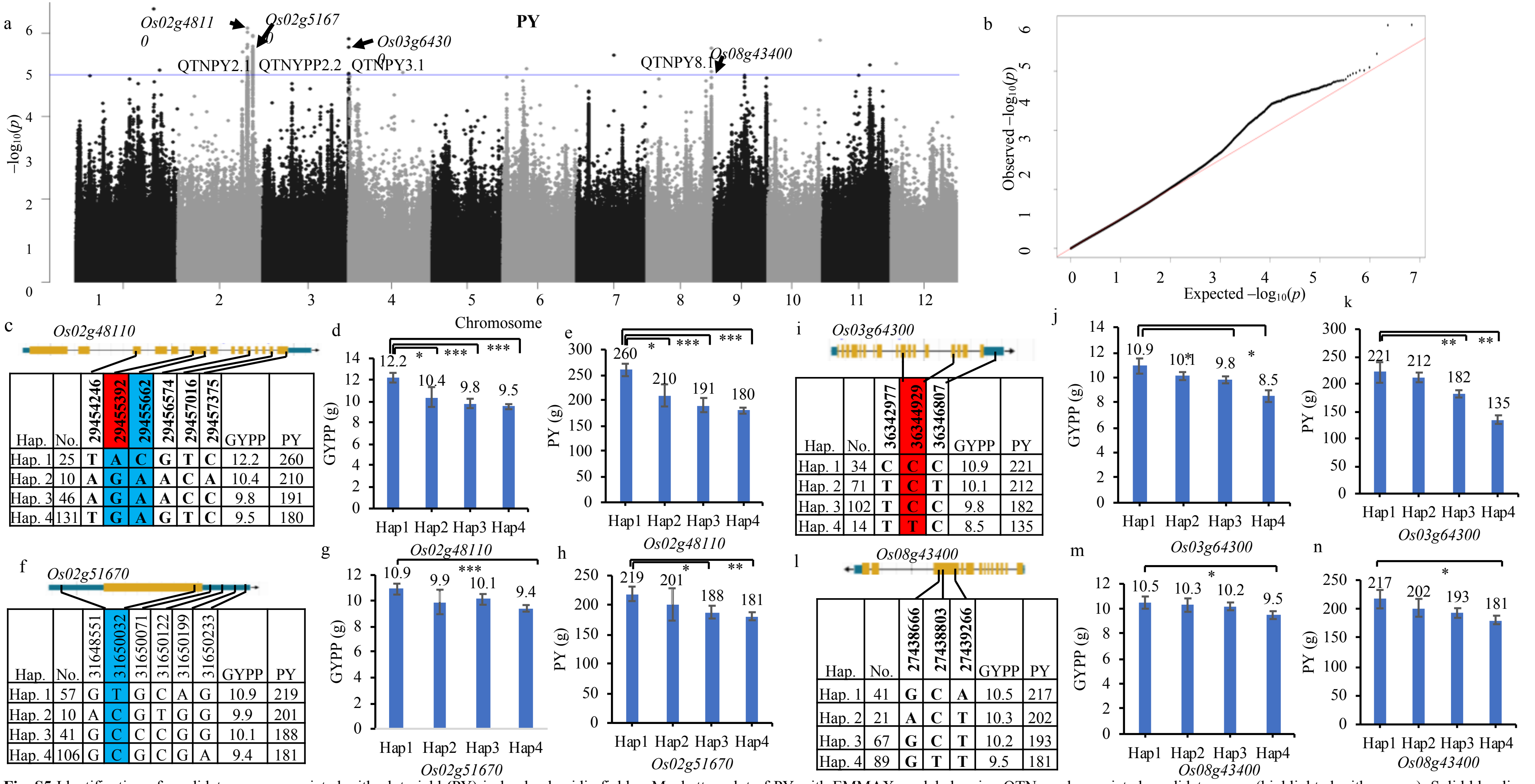


**Fig. S5** Identification of candidate genes associated with plot yield (PY) in lowland acidic field. **a** Manhattan plot of PY with EMMAX model showing QTNs and associated candidate genes (highlighted with arrows). Solid blue line depicts the threshold *P* value for peak SNPs/QTNs. **b** Q-Q plot for PY. Gene structure of candidate genes- **c** *Os02g48110*; **f** *Os02g51670* and **i** *Os03g64300* and **l** *Os08g43400* with haplotype analysis of peak SNPs. Orange, blue and white colour represent exons, UTR and introns, respectively. Nonsynonymous SNPs are in bold; blue and red-coloured columns represent peak, and deleterious SNP (SIFT score < 0.05), respectively. Average value for a particular haplotype (hap.) for GYPP and PY along with number of genotypes (no.) is indicated. Phenotypic variation among haplotypes for GYPP and PY with significant values (*t* test) indicated by * ($P < 0.05$), ** ($P < 0.01$) and *** ($P < 0.001$), respectively for candidate genes- **d** and **e** *Os02g48110*; **g** and **h** *Os02g51670*; **j** and **k** *Os03g64300*; **m** and **n** *Os08g43400*

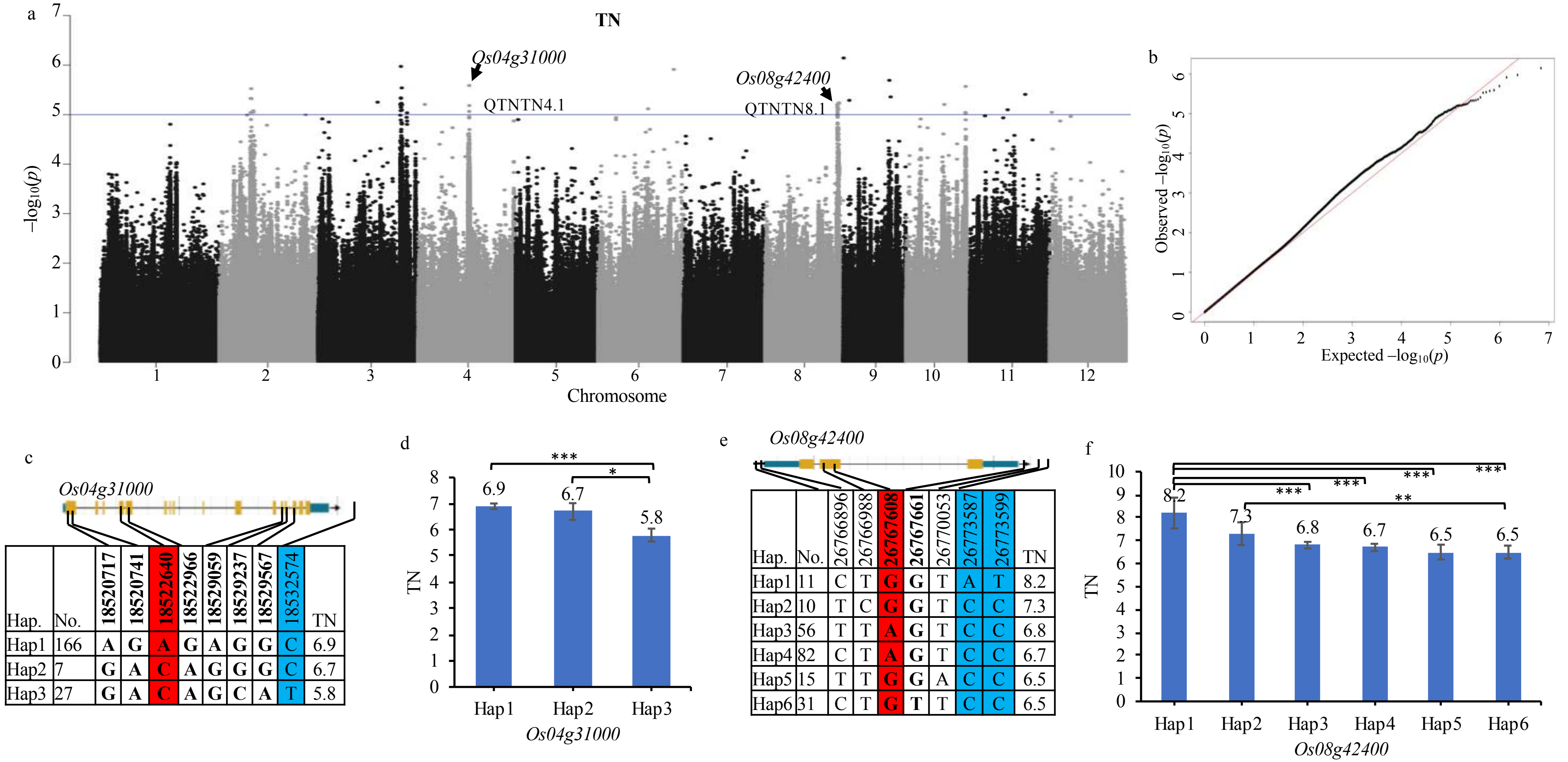


**Fig. S6** Identification of candidate genes associated with TN. **a-b** Manhattan plot and Q-Q plot of TN with EMMAX model showing QTNs and associated candidate genes (highlighted with arrows). Gene structure of candidate genes- **c** *Os04g31000*; **e** *Os08g42400;* with haplotype analysis of peak SNPs. Orange, blue and white colour represent exons, UTR and introns, respectively. Nonsynonymous SNPs are in bold; blue coloured columns represent peak SNP. Average value for a particular haplotype (hap.) for TN along with number of genotypes (no.) is indicated. **d, f** Phenotypic variation among haplotypes for TN with significant values (*t* test) indicated by * ($P < 0.05$), ** ($P < 0.01$) and *** ($P < 0.001$), respectively for candidate genes
